# Re-thinking Linkage to Care in the Era of Universal Test and Treat: Insights from Implementation and Behavioral Science for Achieving the Second 90

**DOI:** 10.1007/s10461-019-02541-5

**Published:** 2019-06-03

**Authors:** Michael E. Herce, Benjamin H. Chi, Rodrigo C. Liao, Christopher J. Hoffmann

**Affiliations:** 1 Division of Infectious Diseases, Department of Medicine, University of North Carolina School of Medicine, 130 Mason Farm Rd. (Bioinformatics), 2nd floor, CB# 7030, Chapel Hill, NC 27599-7030 USA; 2 Division of Global Women’s Health, Department of Obstetrics & Gynecology, University of North Carolina School of Medicine, Chapel Hill, USA; 3 Department of Health Behavior, UNC Gillings School of Global Public Health, Chapel Hill, USA; 4 Department of Medicine, Johns Hopkins University School of Medicine and School of Public Health, Baltimore, USA

**Keywords:** HIV, Linkage to care, Test and treat, 90-90-90, Implementation science

## Abstract

To successfully link to care, persons living with HIV must negotiate a complex series of processes from HIV diagnosis through initial engagement with HIV care systems and providers. Despite the complexity involved, linkage to care is often oversimplified and portrayed as a single referral step. In this article, we offer a new conceptual framework for linkage to care, tailored to the current universal test and treat era that presents linkage to care as its own nuanced pathway within the larger HIV care cascade. Conceptualizing linkage to care in this way may help better identify and specify processes posing a barrier to linkage, and allow for the development of targeted implementation and behavioral science-based approaches to address them. Such approaches are likely to be most relevant to programmatic and clinical settings with limited resources and high HIV burden.

## Resumen

Para vincularse a la atención médica con éxito, las personas que viven con VIH tienen que negociar una serie de pasos complicados desde el diagnóstico del VIH hasta las interacciones primeras con los sistemas y proveedores de cuidado y tratamiento del VIH. A pesar de la complejidad involucrada, muchas veces el vínculo con la atención médica es demasiado simplificado y se considera como un solo paso de remisión. En este artículo, ofrecemos una nueva definición operativa de la vinculación con la atención médica, adaptada a la era actual de prueba y tratamiento universal que establece la vinculación a la atención médica como su propio camino matizado dentro de la cascada más amplia del tratamiento de VIH. Conceptualizando la vinculación a la atención médica de esta manera puede ayudar a identificar y especificar mejor los procesos que dificulten la vinculación a la atención médica, y permitir el desarrollo de estrategias dirigidas y basadas en la ciencia de implementación y de compartamiento para abordar esos procesos obstructivos. Es probable que estas estrategias sean más relevantes para contextos programáticos y clínicos con recursos limitados y una alta prevalencia de VIH.

## Introduction

Global calls to realize the UNAIDS 90-90-90 targets have been transformative, mobilizing governments and the international community to ensure: (1) 90% of people living with HIV (PLHIV) know their HIV status; (2) 90% of PLHIV who know their status start and sustain antiretroviral therapy (ART); and (3) 90% of PLHIV on ART achieve viral suppression. At the heart of these targets is a global effort to “treat all” PLHIV irrespective of CD4 count or clinical stage, a strategy known as universal test and treat (UTT). When implemented at scale, UTT can promote universal access to ART and lead to profound individual and public health benefits [1–6]. As a result, UTT has been codified in countless country treatment guidelines since WHO endorsement in 2015 [7, 8], and is currently being implemented in many clinical and programmatic settings globally in pursuit of the first and second 90s.

Despite this global progress, efforts to achieve the second 90 have been hampered by persistent challenges with linkage to care (LTC), the pathway from HIV diagnosis to initial engagement with HIV care and treatment. In 2017, 41% of all PLHIV globally, and 21% of those aware of their HIV status were not receiving ART [9]. Recent pragmatic trials from sub-Saharan Africa highlight the extent of the LTC problem in those countries most heavily burdened by HIV. Two cluster randomized trials of treatment as prevention (ANRS 12249 [TasP] and HPTN 071 [PopART]) reported only 42–48% of adults linking to care after testing HIV-positive despite receiving a variety of linkage support services [10, 11]. Other work—mostly from before the UTT era—has identified important barriers and facilitators to LTC in low and middle-income countries (LMICs) [12–15], and provided behavioral models to conceptualize HIV care utilization and LTC [16–18]. What appears less explored and, thereby, incompletely understood, are the processes integral to LTC in settings implementing UTT. In this paper, we present a conceptual framework that articulates the individual processes comprising LTC, and the service delivery approaches and health-seeking behaviors needed to successfully achieve LTC in the UTT era. Our goal is to provide a clear and operationally relevant description of these processes to support the implementation of evidence-based approaches to improve LTC, with particular focus on clinical and programmatic settings in LMICs highly burdened by HIV/AIDS.

## A New Approach to Framing Linkage to Care

Successful LTC should reflect more than the first visit to the ART clinic, the first ART prescription [19], or a simple handoff between HIV testing and treatment providers. Instead, a programmatically meaningful definition of “full” LTC should include, at a minimum, that a newly HIV-diagnosed individual has received health communication regarding the HIV test result with post-test counseling, has registered for care, has been evaluated for ART, has received ART education (from a clinician or counselor), and has been dispensed and initiated ART. In our framework, we also include a return follow-up visit—after the initial ART dispensation—as a step in the LTC definition. This is to provide some assessment of linkage beyond a single (and potentially fleeting) encounter with the health care system. Notably, reports of patients who collect ART for the first time but never return for follow up range from 15% to over 30% across a range of settings [20–22], calling into question whether these patients were ever truly linked to care. Including a follow-up contact with the health care system—either in the facility or the community—enhances the assessment of linkage as being fully connected or linked into care versus having simply passed by an ART prescriber, but not having committed to continuing care or taking medications.

Another important consideration for a programmatically useful LTC definition is the time interval from testing HIV-positive to initiating ART care and attending follow-up. In routine practice, this time can range from minutes, to days, to weeks, or even months. A variety of time intervals have been used in the literature and for programmatic reporting, mostly focused on time from testing to one of the following steps: enrollment, first clinical evaluation, ART initiation, or baseline laboratory testing [23, 24]. Historically, the time interval used (e.g., 30, 60, or 90 days) has reflected programmatic needs resulting from routine data collection activities, funder reporting requirements, limited program resources, or other pragmatic considerations. Emerging data suggest that applying 1 month as a “deadline” for establishing LTC may be a programmatically useful time point to predict future viral suppression [24] and to identify “unlinked” individuals who may benefit from more intensive LTC services [25]. For example, in Uganda, 53% of newly HIV-diagnosed patients linked to care by 1 month—defined as registering for ART at a facility—with that figure increasing only slightly to 56% by 3 months post-diagnosis [21]. After 1 month, the incremental likelihood of patients linking to care increased only marginally [24]. Passively waiting beyond a few weeks, let alone 1 month, for a newly diagnosed HIV-positive individual to link to care may undermine a program’s ability to successfully identify, and then trace, find, and engage, individuals who have not yet achieved LTC.

**Fig. 1:**
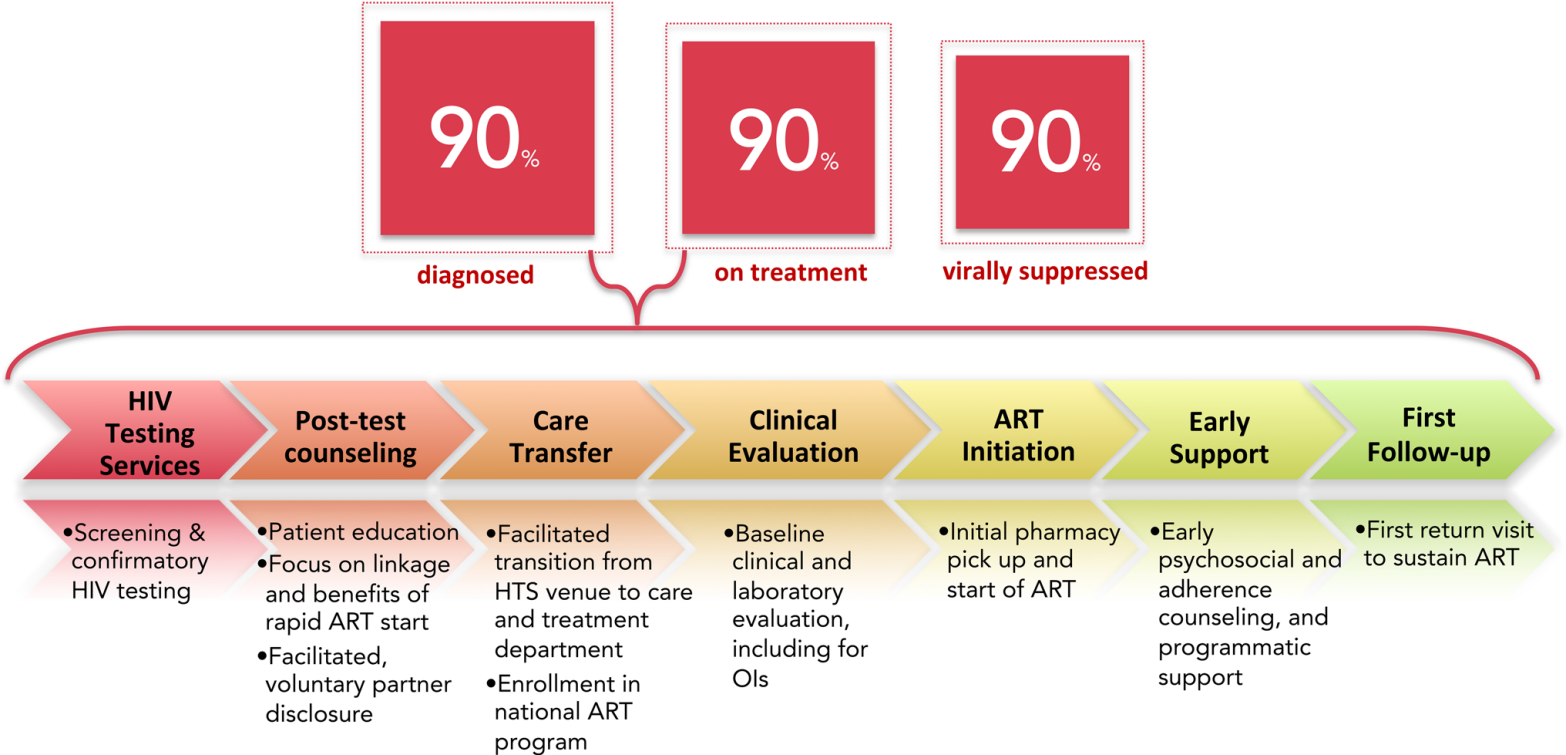
Linkage to care pathway representing the steps necessary to ensure full linkage to care and treatment

In the time from testing HIV-positive, to being dispensed and initiating ART, to the first follow-up visit, there are multiple steps that should occur. We summarize these steps as part of a pragmatic operational definition of LTC, which frames LTC as its own pathway within the larger HIV cascade (Fig. 1). The steps include appropriately educating or counseling a patient, facilitating transfer to the care and treatment department, investigating for co-morbid infections and assessing safety of the planned ART regimen during clinical evaluation, initiating ART and dispensing other medications, providing early support, and completing a first follow-up visit. Ideally, the LTC pathway should take no more than a few weeks to complete all steps, including same-day or rapid ART initiation (within 7 days of diagnosis) in line with WHO guidelines [26], and a first return visit. The LTC pathway is informed by the social ecological model [27, 28] and the conceptual model of implementation research [29, 30], and thus reflects a complex set of processes, each involving dynamic interplay among multiple health system-, facility-, and patient behavior-level factors (Table 1) [12].

**Table 1:** Barriers and health system-, service delivery-, and patient behavioral factors influencing linkage to care

Linkage to care pathway step & relevance		Factors			
		System-level	Health service delivery	Patient behavioral	Barriers
HIV testing services	Counseling and testing services necessary to accurately and safely establish an HIV diagnosis	Setting (facility, venue, community, home, etc.); maintaining test kit supply chain	Access; provider attitude toward clients; provider incentives; quality assurance and control mechanisms	Motivation for testing; believing and acting upon test results	Access; test kit quality control; ensuring confidentiality and privacy; preventing inadvertent disclosure
Post-test counseling	Educating patient about HIV/AIDS and implications of the diagnosis for the patient, their partner(s), and their family; emphasizing benefits and availability of ART; discussing importance of LTC	Adequate training, support, and remuneration for counselors	Counseling technique, focus, and content	Engagement and trust in counselor	Adequate counseling space; effective counselor supervision and training
Care transfer	Robust, facilitated transition between testing and treatment departments, ultimately culminating in registration in the national HIV program and meeting treatment provider	Complexity of navigation to care registration	Patient-centered processes; facilitation of transfer/patient accompaniment	Perception of value of HIV care; perception of costs of attending care	Patient perceptions of time and value of HIV care; patient readiness to accept treatment and initiate ART
Clinical evaluation	Initial medical review, including comprehensive history and physical exam, laboratory evaluation, investigation for co-morbid infections and assessment of safety of planned ART regimen	Number of steps or visits required; adequate infrastructure and human resources for health	Integrity of laboratory testing and processes for returning results	Patient capacity (mediated by burden of illness)	Added burden from multiple clinical encounters which may require enduring long queues; clinic space; lab infrastructure; limited human resources and provider training
ART initiation	Patient ART readiness assessment and first ART dispensing	ART supply chain and logistics	Complexity of dispensing ART	Consideration of the relevance of ART and potential side effects	Patient “not ready” to start ART; internal and external stigma
Early support	Psychosocial counseling, often focused on adherence and “positive living,” and other support to lower barriers to care engagement, including voluntary facilitated partner disclosure	Patient-centered support versus health system-centered	Availability of peer supporters, support groups, and counselors; identifying individuals who do not return for support; Adherence counseling technique, focus, and content	Overcoming concerns of disclosure and stigma to engage in available support services	Patient not identifying value; unavailable or insufficiently resourced support services; weak connections between facility- and community-level services
First follow-up	Return visit with the treatment provider to confirm and support taking ART and continuing HIV care	Goals and timing of follow-up	Availability of health workers; capacity of facility for additional patient visits	Value of follow-up to patient	Establishing systems for scheduling early follow-up and reminding patients

### HIV Testing Services

The context and modality of HIV testing services (HTS) and the availability of facilitated LTC services (e.g., accompaniment to the first clinic visit) have important effects on the population reached and the proportion of those testing positive who successfully link to care. Often, this is an inverse relationship. Thus, while community, mobile, or home-based testing may reach a population less likely to undergo testing, these approaches may be associated with lower LTC than facility-based testing when offered without facilitated linkage strategies [31]. For example, in a meta-analysis of 12 studies examining home- and campaign-based HTS, only 26% (95% confidence interval [CI] 18–36%) of newly HIV-diagnosed individuals visited an ART clinic compared to 61% with facility-based testing (95% CI 39–71%) [31]. In contrast, when combined with facilitated linkage interventions, the same meta-analysis suggests that home-based HIV testing and community-based campaign testing can support LTC (defined as a first visit to the ART clinic) that is excellent (95%, 95% CI 87–98%) [31]. Linkage to care following community HTS may be contextually dependent [31]. For instance, in Malawi, just 56% of newly HIV-diagnosed adults realized LTC—defined as accessing confirmatory testing and clinic-based HIV care—following implementation of a novel community-based HIV self-testing strategy [32]. Additional studies are needed to further evaluate LTC after other innovative HTS approaches (e.g., following couples-based testing).

### Post-test Counseling

Post-test counseling has long been a cornerstone of HTS; however, its effectiveness for facilitating LTC has not been rigorously evaluated. Many existing practices for post-test counseling provide HIV-related information that may only be tangentially related to LTC [33, 34]. For example, many national post-test counseling guidelines have historically emphasized healthy lifestyle practices and prevention of HIV transmission through condom use or status disclosure. Until very recently, less attention has been paid to LTC, immediate ART initiation, and messages around achieving an undetectable viral load (e.g., undetectable = untransmittable) [34]. In the UTT era, there is a need to determine the optimal counseling communication technique and content, and the effectiveness of post-test counseling for supporting LTC [35]. In this regard, applying health belief and transtheoretical health behavior models may help adapt existing post-test counseling approaches to enhance LTC [28, 36, 37]. Approaches informed by both models may help counselors better provide client-centered counseling and align the value of LTC with patient goals and values. This may guide a patient in overcoming specific barriers to LTC, and could be particularly important for asymptomatic patients or those with high levels of fatalism or denial.

Longitudinal or “follow-up” counseling that focuses on LTC and occurs subsequent to HTS (and post-test counseling) has been evaluated in multiple studies, yielding modest LTC improvements in South Africa (38% vs. 31%) and Uganda (51% vs. 33%) when compared to standard care [25, 38]. Follow-up counseling generally encourages patients to access care and initiate ART, and is delivered over multiple counseling sessions that explicitly emphasize the importance of early LTC. Importantly, such longitudinal counseling typically takes a client-centered approach to address individual concerns and needs [39]. This process may not be an essential step in the LTC pathway for all patients, but may be important to achieve LTC for a select group. For example, follow-up counseling can be targeted toward patients in denial of an HIV diagnosis, those who do not accept ART on the day of testing, or PLHIV who fail to initiate ART within a specified time frame [40]. If, where, and how follow-up counseling should be positioned within the LTC pathway and existing HIV service delivery platforms needs further evaluation.

### Care Transfer from Testing to Treatment

After HIV testing and counseling, a structured process should take place in which the newly diagnosed patient seamlessly transfers between testing and care settings. This transfer may take several forms. The simplest scenario occurs when the HTS provider is also the ART prescriber. There may also be direct transitions to ART assessment and dispensing following post-test counseling when HTS and ART services are co-located and ART providers are immediately available to assess a patient. Even in this case, multiple challenges may complicate a seemingly simple process, including time constraints for the provider or patient, the patient’s desire to receive HIV care elsewhere (i.e. transfer openly or “silently”), high patient volumes competing for the provider’s time, or local practices regarding ART preparedness and safety assessments. Despite such challenges, ART initiation during the HTS encounter has been delivered successfully in rural Lesotho and in antenatal care in multiple LMICs [22, 41, 42]. In other settings, HTS team members may accompany a patient to initiate ART at a facility located elsewhere from where testing occurred [43, 44]. Such personalized accompaniment from the testing to the treatment venue warrants further study as a strategy to strengthen care transfer. Common to each of these approaches is a client-centered approach to service delivery. Considering the client needs before health system convenience is likely to improve success with all ART initiation approaches. Overall, success with facilitating care transfer from HTS to ART providers has been demonstrated by reducing the number of, and time required for, steps in the transfer process [22, 44–46].

### Clinical Evaluation

Clinical evaluation is required prior to ART initiation. The goals of this evaluation include: inspiring confidence in ART care; obtaining a complete medical history, including ART history to identify and optimize care for any returning patients re-initiating treatment after prolonged ART interruption or care disengagement (e.g., the so-called “side door” to the HIV cascade) [47]; conducting baseline laboratory testing to assess for co-morbid conditions (e.g. chronic kidney disease) and to determine the severity of HIV disease (e.g., baseline CD4 cell count testing); and screening for sub-clinical opportunistic infections (OIs) and other potentially life-threatening conditions that could be exacerbated by the planned ART regimen, such as cryptococcal meningitis and tuberculosis (TB). This evaluation can be a barrier to LTC if it necessitates multiple clinical contacts and excessive delays [13], as ART can be successfully initiated prior to having laboratory results in LMICs [48]. Local context, including the availability of point-of-care (POC) testing, may influence the prioritization, timing, and speed of baseline laboratory evaluation during ART initiation [49, 50].

As the leading cause of morbidity and mortality among PLHIV in LMICs, assessment for TB is an essential part of the clinical evaluation, but one that may add complexity to the LTC pathway due to the lack of a simple, rapid, and affordable POC diagnostic tool in most clinical settings [26, 51]. Reducing or eliminating the patient-level complexity and duration of evaluation for TB and other OIs may improve LTC [52]. Rapid near POC testing with Xpert MTB/RIF [53], POC testing with TB-LAM [54], co-locating TB and HIV diagnostic and treatment services [55], and telephonic delivery of test results to providers and patients (before or after ART initiation) have all shown promise for streamlining TB and HIV care integration [56].

Antimicrobial prophylaxis can prevent TB and other co-morbid infections, does not adversely affect viral load suppression despite the added pill burden [57], and should be smoothly integrated within ART care to facilitate LTC rather than create additional barriers. While isoniazid preventive therapy and cotrimoxazole are the most commonly prescribed, recent evidence supports the use of shorter course TB preventive regimens [58, 59], as well as some combination of fluconazole, azithromycin, and albendazole provision in patients with advanced immunosuppression [57]. Seamlessly integrating OI preventive therapy and new laboratory testing modalities within the baseline clinical evaluation may require adopting human-centered design approaches to ensure maximally efficient patient care services responsive to clinic workflow, available human resources for health, and other health system constraints [60].

### ART Initiation

There are multiple potential benefits of initiating ART on the same day as diagnosis, including promotion of early LTC and reduction in the number of health service contacts prior to ART initiation [44, 61]. In Lesotho, for example, offering home-based ART initiation on the day of testing increased LTC by 25.6% (95% CI 13.8–36.3%) at 3 months compared to usual care [22]. Health system encouragement of “same day” treatment may also serve to reinforce the health benefits of early ART, just as is done for many other infectious diseases. Disadvantages of starting ART on the day of testing may include increased risk of failing to attend a first follow-up visit and resulting failure to establish full LTC. If ART is started without proper patient preparation, it is possible that its use may be sporadic, which in turn could lead to HIV drug resistance, especially with regimens containing efavirenz or nevirapine. Patient-centered approaches to measuring ART readiness—as has been done in some settings [61]—may help identify patients requiring additional counseling and support to ensure long-term adherence. These approaches likely need further refinement to be generally useful and should not unduly delay ART initiation among those who are ready to start and likely to remain adherent over time.

### Early Support

Careful consideration must be given after ART initiation to services to help patients adjust to new realities of managing a chronic illness. Insights from social cognitive theory suggest that there is a dynamic relationship between a patient’s behavior toward managing their illness and their environmental and physiologic milieu, which interact in myriad ways to affect longitudinal engagement in care and, ultimately, health outcomes [28]. Empowering patients with skills to positively influence their environment, build self-efficacy, and overcome personal and structural barriers to care can help patients develop positive support networks, fully engage with healthcare providers, and take their medications regularly [62].

Pragmatically addressing the social determinants of health, including poverty, malnutrition, and social isolation through socioeconomic interventions, patient-enabling strategies to facilitate service access, nutritional supplementation, and community health worker or peer navigation services may also assist some patients to more fully establish care during the early stages of their treatment and develop a stronger bond with the care system [63, 64]. How early peer-based support may generate other downstream programmatic benefits, such as by promoting voluntary partner disclosure for HIV index testing or creating patient demand for viral load testing, require future investigation. Group support models, such as community adherences groups and patient ART initiation “clubs”, and other differentiated service delivery (DSD) models may decrease barriers and opportunity costs of care while potentially increasing value of care engagement. Such approaches may prove feasible and effective for patients in some settings [65, 66]. DSD models initially designed for stably in-care patients should be further studied and adapted to the needs of new and returning patients to better support them as they face the first days and weeks of (re-)treatment.

Underlying many early support interventions is a focus on providing adherence counseling. Adherence counseling can be delivered in group, community, or facility settings by lay counselors, peers, community health workers, nurses, or clinicians, depending on the local context. While multiple adherence counseling sessions have been required for patients to start ART and promote adherence in many programs (e.g., 3 counseling sessions over 3 weeks before ART can be initiated), additional research is needed to elucidate the optimal form and timing of adherence counseling within the LTC pathway. Notably, there are few data on the effectiveness of, or best practices for, adherence counseling.

### Early Follow-Up

Early follow-up shortly after ART initiation is thought to be important. This is a period during which ART adherence can be re-enforced, ART side effects managed (prior to a patient self-discontinuing treatment), and patient strategies introduced for managing internalized stigma and coping with living with HIV. While the exact timing for optimal early follow-up is unknown, operational data from routine care settings suggest that scheduling a first follow-up visit a few months after ART initiation is too long to wait and may undermine patient care engagement [21]. Moreover, the ideal modality for early follow-up (e.g., provider-patient clinic encounter, pharmacy visit, community- or home-based visit, group meeting, or phone call) is also unclear. Finally, while interventions such as flexible clinic hours and text message appointment reminders hold promise for facilitating early ART use [67], optimal strategies to promote early follow-up and how early follow-up influences later retention in care are areas of uncertainty.

## Monitoring and Measuring Linkage to Care

A universal operational definition for LTC responsive to the UTT era is urgently needed to allow comparison of LTC outcomes over time within and across programs. We propose a programmatic definition for “full” LTC that presents the proportion of newly diagnosed HIV-positive individuals who successfully register in the national treatment program, undergo baseline clinical and laboratory evaluation, collect a first ART prescription, initiate ART, *and* return for a first follow-up visit—all within a well-specified time frame (e.g., no more than 2 to 4 weeks from HIV diagnosis). Such a definition may be applied at an individual level to identify patients who have not achieved full LTC or at a program level to prompt corrective action when a program’s LTC “rate” fails to meet a pre-specified target. To be most effective, LTC data must be tracked for individual patients as they progress through each step in the LTC pathway, with a particular focus on ART initiation [19], so providers and program managers can identify areas of attrition and develop ways to address them. The use of a paper-based linkage register that captures every new HIV diagnosis within a facility’s catchment area and documents patient HTS, ART, and laboratory identifying numbers from disparate data sources in one data recording tool may be a low-cost approach for improving individual-level LTC reporting. Newer strategies to link diverse HIV program data, such as biometrics and SIM chip- or barcode-enabled patient cards encoding a national health identifier, merit further evaluation and scale up [68]. Using such individually linked health information across data platforms is the most promising strategy to provide true patient-level descriptions of LTC. Critically, these linked data need to identify in as real-time as possible: (1) patients who have not fully linked and may benefit from a specific enhanced LTC strategy; and (2) clinic-level trends to guide quality improvement activities. Implementing partners and funding agencies should move away from reporting uncoupled cross-sectional estimates of LTC (e.g., total number of PLHIV newly initiating ART divided by the total number of new HIV diagnoses in a reporting period), which do not track individual patients along the LTC pathway and may overestimate rates of successful linkage. Instead, employing longitudinal information, programs should adopt sequential time-defined patient-level “cohort” reporting, which can be used to assess LTC changes over time and to monitor LTC in populations who may be less likely to achieve full LTC (e.g., adolescents) [69]. Where universal national health identifiers are not available, cohort-based reporting within a geographic area (e.g., district or province) may be assisted by electronic health record data capture at all service entry points along the LTC pathway [70] and the use of probabilistic patient matching algorithms to identify silent transfers, “side door” LTC pathway entry [47], and minimize duplicate patient records [71].

## Conclusion

Successful LTC requires a patient to navigate a series of processes, from testing HIV positive through post-test counseling, care transfer, clinical evaluation, ART initiation, and initial follow-up. Identifying the process that is the greatest barrier to successful LTC—at health system, service delivery, or patient behavior levels—is a critical first step. Once identified, the barrier can be specified, characterized, and measured to spark program improvement. With this clear understanding in hand, targeted and context appropriate health system strategies or patient-level interventions can be designed to address the deficient process or processes within the LTC pathway. Critical examination of LTC as its own pathway will help to illuminate how and where intervention are, and are not, working and enable the design of new implementation strategies to achieve the second 90.
